# Intervention description is not enough: evidence from an in-depth multiple case study on the untold role and impact of context in randomised controlled trials of seven complex interventions

**DOI:** 10.1186/1745-6215-13-95

**Published:** 2012-06-28

**Authors:** Mary Wells, Brian Williams, Shaun Treweek, Joanne Coyle, Julie Taylor

**Affiliations:** 1 School of Nursing and Midwifery, University of Dundee, 11 Airlie Place, Dundee, DD1 4HJ, UK; 2 Nursing, Midwifery and Allied Health Professions Research Unit, Iris Murdoch Building, University of Stirling, Stirling, FK9 4LA, UK; 3 Population Health Sciences, School of Medicine, University of Dundee, The Mackenzie building, Kirsty Semple Way, Dundee, DD2 4BF, UK; 4 Social Dimensions of Health Institute, University of St Andrews and University of Dundee, 11 Airlie Place, Dundee, DD1 4HJ, UK; 5 National Society for the Prevention of Child Abuse and Neglect (NSPCC), Weston House, 42 Curtain Rd, London, EC2A 3NH, UK

**Keywords:** RCT, Case study, Context, Complex interventions

## Abstract

**Background:**

A number of single case reports have suggested that the context within which intervention studies take place may challenge the assumptions that underpin randomised controlled trials (RCTs). However, the diverse ways in which context may challenge the central tenets of the RCT, and the degree to which this information is known to researchers or subsequently reported, has received much less attention. In this paper, we explore these issues by focusing on seven RCTs of interventions varying in type and degree of complexity, and across diverse contexts.

**Methods:**

This in-depth multiple case study using interviews, focus groups and documentary analysis was conducted in two phases. In phase one, a RCT of a nurse-led intervention provided a single exploratory case and informed the design, sampling and data collection within the main study. Phase two consisted of a multiple explanatory case study covering a spectrum of trials of different types of complex intervention. A total of eighty-four data sources across the seven trials were accessed.

**Results:**

We present consistent empirical evidence across all trials to indicate that four key elements of context (personal, organisational, trial and problem context) are crucial to understanding how a complex intervention works and to enable both assessments of internal validity and likely generalisability to other settings. The ways in which context challenged trial operation was often complex, idiosyncratic, and subtle; often falling outside of current trial reporting formats. However, information on such issues appeared to be available via first hand ‘insider accounts’ of each trial suggesting that improved reporting on the role of context is possible.

**Conclusions:**

Sufficient detail about context needs to be understood and reported in RCTs of complex interventions, in order for the transferability of complex interventions to be assessed. Improved reporting formats that require and encourage the clarification of both general and project-specific threats to the likely internal and external validity need to be developed. In addition, a cultural change is required in which the open and honest reporting of such issues is seen as an indicator of study strength and researcher integrity, rather than a symbol of a poor quality study or investigator ability.

## Background

The detailed reporting of both the operation and findings of intervention-based studies is essential to inform effective decision making and thus lead to real improvements in healthcare provision. Decision makers, whether at policy or practice level, need sufficient information in both domains to answer four questions: What level of benefit was achieved in the research? Are the findings valid and reliable? Is the intervention sufficiently defined as to allow it to be replicated? If replicated in other places, would similar benefit still be achieved?

For ‘simple’ interventions such as pharmaceutical products (those which are easy to define, have few components and for which the active ingredients are known) researchers face relatively few problems in providing sufficient information to help answer these questions. For example, benefit can be defined in terms of confidence intervals around primary and secondary outcome measures; reliability and validity can be assessed through adherence to a pre-set list of methodological criteria; the intervention can be defined in terms of dose and frequency; and it is reasonable to assume that a similar benefit will be obtained if replicated in a similar population, irrespective of place.

However, policy makers and practitioners are increasingly asked to make judgements regarding complex rather than simple interventions; in these cases an array of new problems arise. Complex interventions in healthcare are built up from a number of components, which may act independently or interdependently although the ‘active ingredient’ is generally difficult to specify [1]. The components usually include behaviours, characteristics of behaviours (for example, frequency, timing), and methods of organising and delivering those behaviours for example type(s) of practitioners, setting and location.

In this paper, we describe potential problems identified in the literature regarding the operation and reporting of randomised controlled trials (RCTs) of complex interventions. While a number of case studies have shown that the context within which intervention studies take place may sometimes challenge the way in which interventions are delivered (particularly with regard to dose, fidelity and reach) [2-4], the focus on a single trial has necessarily limited their ability to identify the diverse ways in which context may challenge the central assumptions of the RCT, the degree to which these occur consistently across different trial or intervention types, or the degree to which this information is known to researchers or subsequently reported.

### The RCT and the challenge of the complex intervention

Randomised controlled trials and meta-analyses remain the Gold Standard for evaluating the effectiveness of interventions and informing guidelines, protocols and policies. The strength and usefulness of the RCT design lies in its power to provide a credible link between cause and effect. However, decision makers need to be able to understand and define the cause. There is potential for the cause to be the intervention itself, elements of the healthcare context within which the intervention is being delivered, elements of the research process that are introduced to that setting (for example, presence of researchers and their operations), or a combination of all three. In other words, it is often difficult to separate the intervention from the context within which it was evaluated.

Common sense suggests, therefore, that RCTs of both complex and simple interventions face these challenges, as both take place in healthcare and experimental contexts which may adapt and evolve, be unpredictable, and involve the interconnected actions of individuals [5]. However, for simple interventions these may pose fewer problems as the intervention is easier to define, easier to separate from context, and those contextual influences that may have the potential to influence the results are easier to separate and standardise [6]. The more nuanced relationship between complex interventions and the healthcare and experimental contexts in which they are situated, poses a greater number of important challenges.

First, the components of a complex intervention may be difficult to define precisely as the distinction between intervention and context is unclear. The rigour that is at the heart of the scientific method embodied in the RCT requires a hypothesis, which includes an *a priori* definition of the intervention (that is, that A will/will not lead to B). However, given that complex interventions may consist of a mix of people, skills, devices, contexts, processes, actions and decisions, developing a definition of ‘A’ is always likely to be problematic, and this has been recognised by the UK’s Medical Research Council (MRC) in their framework for the development and evaluation of complex interventions [7]. In practice, a single approach to definition may not be possible. Indeed, authors have pointed out that complex interventions require flexibility in their definitions, so that instead of defining and standardising them by ‘form’, they should be defined by ‘function’ [8], with clear indication of whether components are ‘fixed’ or ‘flexible’ [9].

Second, even where interventions can be defined and separated conceptually from their healthcare contexts, those elements of context that might influence trial operation or outcome may not be straightforward to identify and may be almost impossible to control or standardise. Indeed some settings may themselves be characterised as complex systems: being multifaceted, experiencing constantly shifting contexts and more similar to a dynamic ecology [10]. The greater the complexity of the intervention, the greater the degree to which an intervention definition blurs into or depends on elements of context for its effectiveness. If the context cannot be fully controlled, then standardisation of a blurred intervention becomes impossible [11].

Third, since defining the intervention and controlling the clinical and experimental context is problematic, it may be difficult to know post facto what precisely led to any change detected in the RCT. Consequently, there is likely to be insufficient information to allow practitioners to make meaningful decisions about whether and how to implement it in their own setting to maximise effectiveness [12-15]. Even pragmatic trials do not fully get around these problems - although they provide more information about real world settings, their heterogeneity may limit the usefulness of their results for specific clinical situations [16,17], and the debate continues as to the merits and pitfalls of explanatory versus pragmatic trials [18]. Understanding the particular contexts in which interventions are evaluated is important for any clinical decision maker, regardless of where the trial sits on the explanatory-pragmatic continuum, as ‘any attempts to extrapolate from study settings to the real world are hampered by a lack of understanding of the key elements of individuals and the settings in which they were trialed’ (ICEBeRG, p5) [19].

If the relationship between intervention and context cannot be fully controlled then it should at least be fully acknowledged and its likely impact reported. This would assist in the interpretation of the results of RCTs [20], the implementation of research [21], and the synthesis of evidence from RCTs of complex interventions [22]. In practice, many studies lack basic information about the trial and clinical contexts [15]. This is perhaps unsurprising, as guidelines for reporting trials, have not, until recently, emphasised the importance of details about intervention components, standardisation and adherence [23,24]. Indeed, these guidelines omit some aspects of interventions that may be important to understanding links between treatment and outcomes, including cultural sensitivity, adaptability and strategies for treatment implementation [25]. Given that inadequate reporting of these issues can undermine judgements about the quality and generalisability of trials, it is important to explore ways in which reporting can be improved and a common language developed.

Although retrospective data collection about trial implementation may be helpful (particularly in detecting unanticipated issues), a more rigorous approach would be to know *a priori* those issues that are likely to threaten the internal validity of the trial and those that may impede the effectiveness of the intervention. Previous research has explored aspects of the context to: design a trial [26]; pilot or understand an intervention [27]; and explain process or interpret the findings of their research [28]. However, the issues and problems identified by this research have not been explored across a spectrum of different trial situations and we therefore do not know whether they are generalisable to other trials of complex interventions.

Consequently, this paper reports a study that moves beyond previous single case studies of complex interventions, and uses a multiple case study approach to explore these diverse issues. Further details of the study including extensive description of methods and wider findings are available elsewhere [29]. In particular, this study seeks to:

· explore, from the perspectives of researchers and practitioners, what goes on ‘behind the scenes’ in randomised trials of complex interventions and establish what information on potential threats to trials is available and known to those running them;

· set out the particular challenges of achieving control and standardisation in a real life setting, and;

· describe key elements of trial environment and indicate how this might affect the implementation of complex interventions.

## Methods

A multiple case study approach was chosen as it explicitly acknowledges the importance of context [30] and is known to be particularly useful for answering ‘how’ or ‘why’ questions through the detailed examination of complex phenomena [31]. The distinct features of the case study approach are the use of multiple sources of evidence and the development and testing of theoretical propositions. Case studies can be exploratory (focusing more inductively on developing theory and theoretical propositions), explanatory (focusing on the more detailed examination and testing of propositions), or a combination of both [32].

The study was conducted in two phases. Phase one informed the design, sampling and data collection within the main study (Table 1). In particular, it attempted to identify potential variables of complexity and establish theoretical propositions that could be investigated and tested in phase two’s trials (Table 2). Phase two consisted of a multiple explanatory case study covering a spectrum of trials of different types of complex intervention. The study was approved by the Tayside Medical Research Ethics Committee, reference number 05/S1401/1.

**Table 1:** Description of intervention in single exploratory case study

**Abbreviated name for trial**	**Aim of trial**	**Setting and wider context**	**Nature of intervention**	**Nature of control intervention(s)**
Phase 1: Single exploratory case				
Aim: To identify potential variables of complexity for and to establish theoretical propositions that could be tested in phase 2				
NLED	To evaluate the impact of a specialist nurse-led model of early discharge following breast cancer surgery on patients, carers and the health service, whilst developing collaborative care pathways that enable a smooth transition between acute and primary care	NHS teaching hospital/cancer centre on two sites (one large university hospital, one smaller district general hospital), with a large geographical catchment area encompassing urban, rural, affluent and deprived communities in North East/ Central Scotland	Nurse-led early discharge - going home with wound drains still *in situ*, one or two days after surgery, with a package of care in place. This group was allocated to one of two nurses from the breast care team who received training from clinical nurse specialists	Usual care - staying in hospital post-operatively until all wound drains were removed (approximately five or six days)

NHS, National Health Service.

**Table 2:** Description of interventions in phase 2

**Abbreviated name for trial**	**Aim of trial**	**Setting and wider context**	**Nature of intervention**	**Nature of control intervention(s)**
Phase 2: Multiple explanatory case study				
Aim: To seek empirical support for the theoretical propositions and assess likely validity across multiple trial types and contexts				
Trial 1: Waiting list	To determine whether patients and their partners who receive an extended home-based programme of pre-cardiac surgery education and support demonstrate equal or greater benefit compared with those who receive a short hospital- based programme	NHS Health Board covering a large geographical area with no cardiac surgery. Contract with a neighbouring Health Board for cardiac surgery. Mixed urban/rural, affluent/ deprived population, with a range of GP practices (single-handed to group practices)	Education and support intervention for patients on waiting list for coronary artery bypass surgery, delivered over six months with alternate monthly home and practice nurse visits	One-off three-hour education and support class delivered in hospital setting for patients in the same target group, soon after joining the waiting list
Trial 2: Information	To evaluate the benefits of a new information leaflet given to parents/guardians when their child is discharged from hospital following a benign febrile convulsion	Paediatric unit in large teaching hospital in Scotland	An information leaflet for parents of children with benign febrile convulsions, designed to meet current standards of written information	An information leaflet for the same target group, not designed to meet current standards of written information
Trial 3: Exercise	To determine the acceptability and effects of an aerobic exercise intervention during radiotherapy for localised prostate cancer on fatigue	Cancer centre in Scotland	Exercise intervention to reduce fatigue in patients undergoing four weeks of radiotherapy for prostate cancer. One to one session with physiotherapist for tailored exercise instruction	A weekly phone call with a clinical nurse specialist. No specific exercise guidance
Trial 4: CBT	To assess the relative efficacy in routine practiceof clinical nurse specialists providing cognitive-behaviour therapy for psychotic patients suffering from chronic, distressing symptoms only partially relieved by medication	Hospital and community psychiatric services in a large Health Board in Scotland	Twenty sessions of cognitive behavioural therapy, delivered over nine months, for patients with medication-resistant psychotic symptoms	Two controls: 1) usual psychiatric care 2) twenty supportive psychotherapy sessions
Trial 5: Guidelines	To determine the effectiveness of a national guideline in improving quality of life in epilepsy sufferers. To evaluate the effectiveness of two different implementation strategies for this guideline	General practices in a large Health Board area in Scotland	Interactive workshops, structured record sheets and specialist nurse facilitation to improve the management of epilepsy through clinical guidelines	Two controls: 1) guideline only sent to GP practices 2) interactive workshops and structured record sheets but no specialist nurse input
Trial 6: Sleep	To examine the impact of disorders of initiating and maintaining sleep on mothers’ mental health. To evaluate the cost effectiveness of a tailored sleep programme, provided by health visitors, in improving the mother’s mental health and the child’s sleep disorder	Fifteen GP practices within a large Health Board area in Scotland parents for children’s sleep	Tailored behavioural intervention programme delivered to disorders by a health visitor over six weekly sessions	Two controls: 1) information booklet only 2) waiting list for programme
Carers	To evaluate the effectiveness of a community mental health nurse (CMHN)-led intervention in supporting carers who look after a person who is diagnosed with schizophrenia. The intervention was compared with support that is normally offered to carers by CMHNs	Two Health Board areas in Scotland. Recruitment also took place through local carer support groups	Supportive intervention to meet the needs of carers of people with schizophrenia, delivered at home over twelve weeks by nurses trained to provide a carer-focused intervention	Supportive intervention to same target group, delivered over twelve weeks by nurses not trained to provide a carer-focused intervention

CMHN, community mental health nurse; GP, general practitioner; NHS, National Health Service.

### Sampling

The case in phase one (RCT of a nurse-led model of early discharge following axillary clearance surgery for breast cancer - the NLED trial) was selected for convenience, with MW being the principal investigator (PI) of the trial [33]. Because the trial had been conducted with the intention of using it as a future case, prospective data had been collected by the PI on the conduct and experiences of the trial staff throughout the course of the trial. For phase two, the National Research Register (NRR) [34] was used to locate a further seven trials of complex interventions representing differing characteristics of complexity identified in phase 1 (see Table 3). Trials varied from an information leaflet for parents of children with benign febrile convulsions to a supportive intervention for carers of people with schizophrenia (Table 2). The decision to sample seven trials was pragmatic, and as the selection was based on the complexity spectrum (Table 3), consistent with theoretical sampling of cases as described by Yin [30].

**Table 3:** The complexity spectrum

**Characteristic**	**Simple**	**Highly complex**
1. Number of components within intervention	One for example, single drug	Several for example, rehabilitation package
2. ‘Dose’ or intensity of intervention	Pre-determined/uniform for example, specified dose drug or pre-prepared information leaflet	Dependent on characteristics or participation of individual for example, exercise intervention
3. Clarity re the components of the intervention	Clearly defined components delivered in a specified order for example, diagnostic procedure	Less well-defined components not delivered in a particular order for example, behavioural intervention
4. Degree of confidence in the ‘active ingredient’	Known ‘active ingredient’ for example, specific medication	Unknown active ingredient even if components are known for example, smoking cessation activity
5. Timing of intervention	Single event for example, one-off procedure	Protracted intervention or multiple time points for example, follow-up care
6. Number of people involved in delivery	One	Several
7. Clarity of responsibility in intervention	Clearly defined	Ill-defined
8. Number of professional groups involved	One	Several
9. Technical or professional skill involved	Minimal skill for example, sending information by post	Highly skilled for example, surgical procedure
10. Human interaction required to deliver the intervention	Little for example, dispensing of a medication	Intervention is dependent on human interaction for example, counselling
11. Setting	Single well-defined setting for example, single therapy room	Cross boundary or multiple settings for example, hospital at home scheme
12. Level of patient involvement or active participation	Low for example, radiotherapy treatment	High for example, self-care intervention or group activity
13. Sphere of impact	Narrow for example, influences individual patients but no impact on or involvement of rest of service	Broad for example, service development intervention with implications for entire service
14. Clarity of main outcome	Clear and obvious outcome for example, cessation of smoking, length of stay following early discharge	Ambiguous outcome(s) for example, improvement in quality of life or patient satisfaction, multiple outcomes that are all important

### Recruitment

In phase one, the nurses most closely involved in the NLED trial were asked to participate in in-depth interviews at various times throughout the trial. In phase two, principal investigators (PIs) were contacted by email or telephone and then sent an information letter if they were interested in participating. All agreed to take part in a one-off interview, and gave permission for research documents to be accessed via the Research and Development Office. Six out of seven trials had recently finished recruiting. For the trial that was still underway, both the PI and research assistant were interviewed.

### Data collection

A total of eighty-four key data sources were used across the two phases. Table 4 provides a summary of data types collected. These included in-depth interviews, focus groups and trial documentation. In phase one, four nurses responsible for delivering the intervention were interviewed prospectively on several occasions during the RCT. Two additional interviews were conducted with the research nurse who was responsible for data collection and the ward manager who was closely involved with the patients who participated in the trial. In phase two, a total of seven principal investigators (PIs) and one research assistant were interviewed. A further three focus groups were conducted, one with three research nurses, another with two nurse members of an ethics committee, and one with four PIs. Thus, four PIs took part in a focus group and were interviewed, while other participants were either interviewed or participated in focus groups.

**Table 4:** Summary of data collection

	**Number and nature of participants/documents**	**Total number of data sources (interviews, focus groups and documents)**
Phase 1: Single exploratory case		
Interviews	Thirteen longitudinal interviews with four breast care nurses:	15
	Three interviewed three times.	
	One interviewed four times.	
	Plus: one interview with a research nurse	
	And one interview with a charge nurse	
Documents	Protocol, ethics application, monitoring reports x 2, final report, Minutes of meetings x 15, field notes of PI and researcher	22
Phase 2: Multiple explanatory case study		
Interviews	Seven principal investigators and one research assistant	8
Documents	Seven trial protocols/proposals	30
	Six ethics forms	
	Five monitoring reports	
	Seven final reports	
	Five published papers	
Focus	One group with three research nurses	9
groups	One group with two nurse members of the ethics committee	
	One group with four PIs	
**Total**		84

PI, principal investigator.

In-depth interviews were conducted with PIs and researchers in their workplaces. The topic guide included questions related to their experience of coordinating the trial; their views of how the intervention had developed and was implemented; and the way in which participants and practitioners responded to the trial. Interviews lasted between 60 and 90 minutes. Multiple trial documents such as proposals, ethics submissions, monitoring and final reports, and publications were also examined, providing a more ‘public’ account of each trial.

### Analysis

The transcripts were analysed using the ‘framework’ approach frequently employed in applied qualitative research [35]. It involved several stages. The first five transcripts were read and initial categories were identified (called familiarisation). These were then compared with concepts in trial documents and the literature. The trial documents provided a means of triangulating the personal accounts of researchers with a more objective record of events. An initial thematic framework was developed and applied to transcripts not involved in the familiarisation process. The framework was then systematically applied to the data sets using a qualitative software analysis package (NVIVO 2) [36]. The analysis was developed further by the creation of charts which cross-linked categories and concepts to generate new meanings. Finally, the original research questions were reconsidered and examined to define concepts, map the range and nature of phenomena, create typologies, find any associations and provide explanations. Interviewing stopped when theory saturation was reached, that is, until the additional data did not add to the developing theory.

Data were analysed as they were collected to ensure that the consultation process was comprehensive. Thus, important issues raised in earlier interviews were fed into subsequent ones, enabling emerging conceptual ideas to be explored in further questioning. Several strategies were also employed to improve the accuracy and validity of the data. Regular meetings between the PI and a senior researcher (BW) were held to check the interpretation of key texts, codes and categories, and new lines of inquiry. Rigour was ensured in the coding process through the involvement of a second coder (JT). Part-way through the analysis, the second coder assigned established codes to ten transcripts and compared these with those of the first. Where differences were identified, these were discussed and further refinement of the meaning of specific codes was established to aid consistency, rigour and credibility of the codes [37,38] in the ongoing analysis. The team also searched for disconfirming evidence for developing theory to ensure transcripts were not analysed selectively.

In phase two, interviews, documents and trial documentation were searched to test and further develop the propositions developed in the exploratory case. This took place in three stages. First, MW examined all documentation to identify detailed events and conceptual sub-domains that contributed to each main theoretical proposition. Second, BW and MW then re-examined all documents and independently identified whether or not there was empirical support for each sub-domain in each individual trial/case; where there was disagreement, this was resolved through discussion. Third, the strength and plausibility of support from the data for each proposition and sub-domain was ranked as absent, suggestive support, or clear support. All ranking was agreed by BW and MW.

## Results

### Phase 1: the exploratory case

Interviews with those involved in the NLED trial revealed that challenges to standardisation of the intervention took place in a dynamic context. The attitudes and behaviour of the nurses changed over the course of the trial, as did organisational factors and research staff, all of which influenced how the intervention was delivered. Three key issues were identified.

1. Changing attitudes towards the intervention among practitioners, participants, and researchers. Changes in skills and confidence of practitioners.

2. The influence of trial participants (patients and family members) on practitioner delivery of and engagement with the intervention.

3. Changes in organisational context, including team members, relationships between staff, and the priorities of the practice setting. Thus, interventions can become more or less of a priority in comparison with other events going on within the healthcare context.

Issues identified in phase one - the exploratory case:

1. Changing attitudes towards the intervention.

Nurses adapted the intervention to meet individual patient needs as they became more familiar with the trial and more confident in terms of the intervention and were less likely to strictly follow the protocol when implementing the intervention. Thus the timing of some aspects of the intervention and the use of standardised assessment forms varied.

"*‘*At the beginning … we were new to this and we wanted to make sure we did it all perfectly. I think now we’ve become more relaxed … and realise although we’re doing the study they’re still individual patients and what’s right for them.’ Nurse 1, fourth interview*.*"

This led to patients receiving different combinations of intervention components such as extra phone calls or visits. The way the intervention was delivered was dependent on the individual nurse:

"‘When you are looking at a small team, it’s very much about the people who are delivering it … a lot of the time I think it’s more related to the nurses’ needs, or time … I don’t think we do standardise the intervention to be honest, it’s really up to the individual nurse as to what they put into that patient and it’s obvious that some people get more than others.’ Nurse 4, fourth interview."

Nurses’ increased familiarity with the intervention also encouraged them to suggest modifications to the package. For example, some nurses felt the specialist breast care (intervention) should shift from the first few days after surgery to several weeks later, when the emotional impact of surgery had ‘sunk in’. This did not happen as it would have changed the intervention, but illustrates how nurses providing the intervention developed their own ideas and preferences, and that these changed.

2. The influence of trial participants.

Patients and carers can also influence what happens within an intervention, for example by interrupting, asking questions, changing the subject or simply just being there:

"‘I think there’s probably been women that I’ve been to see that when I think about it now that would’ve probably opened up a bit more but didn’t have the opportunity to because someone was around, the carer was around.’ Nurse 2, third interview."

Patients might also prioritise certain aspects of the intervention above others, and thus influence which components were delivered. One nurse complained of her patients only wanting to talk about their wound drains.

"‘I mean their main concern is the drain … they are desperate to tell me about the drain, you need to say right, ok then, so the drain is fine … let’s talk about how everything else has been.’ Nurse 3, first interview."

Other patients stressed what they felt were critical aspects of intervention, so that the information they gave would be used to benefit of other patients in the future.

3. Changes in organisational context.

"‘Because she knew she was in the study, she kept saying “I’ll just tell you because it might help somebody else”, she has always got this list, list of things she wanted to tell me because it might help somebody else.’ Nurse 1, first interview."

The demands of the organisation, and the perceptions and needs of other staff affected the delivery of the intervention. Minutes of study progress meetings show that the time spent on the intervention depended on practicalities such as travelling to patients’ homes. In severe weather, the breast care nurses gave responsibility for most of the intervention to district nurses who were able to visit patients more easily. However, breast care nurses noticed when they visited together, the district nurses would steer the intervention towards areas unrelated to the intervention. Staff changes during the trial impinged on the way in which the intervention was delivered. Two designated breast care nurses left their posts after the first year of the study and the workload was passed to the two senior nurses, who had other responsibilities. Minutes of meetings record that the senior nurses found the intervention increasingly burdensome and the research nurse describes how their enthusiasm for the intervention waned:

"*‘*The difficulty is because they have lost one member of staff, it is only the two of them and they work five days a week as it is and they must just think they don’t want to do it … and I can sympathise.’ Nurse 4, fourth interview."

Progress reports and meeting minutes repeatedly highlighted organisational factors, which altered the context in which the intervention was delivered and may have introduced new variables. One was the opening of a new five-day ward approximately six months into the trial. This meant that patients in the intervention group were often admitted to the five-day ward, rather than to the original breast surgery ward, to which the control group were admitted. A shortage of anaesthetists also led to numerous cancelled operating lists, which caused organisational difficulties for the implementation of the trial because patients were not admitted as planned.

It was clear from participants’ perspectives that complexity-related threats to internal and external validity existed within and between three key contexts: personal (attitudes and behaviours of staff, patients and family members), organisational (local service delivery structures including organisational and professional roles and responsibilities), and trial (the implementation of the methods of the trial itself on top of the organisation of ‘usual care’). Given this tripartite classification, it became clear that issues could be broken down further to produce a broader list of variables that might define the varying scale and nature of complexity of an intervention and its context. This ‘complexity spectrum’ is shown in Table 3.

Three broad theoretical propositions were generated from the findings of phase one, which appeared to hold for the single exploratory case and could be then tested against a spectrum of further studies of interventions varying in scale and type of complexity.

Proposition 1: The context within which complex interventions take place is crucial to understanding how the particular complex intervention works and whether it will be generalisable to other settings.

Proposition 2: Complex interventions evolve during the process of a trial. Perceptions, roles and practices change over time and have a bearing on the delivery of the intervention.

Proposition 3: If complex interventions comprise of different dimensions, are influenced by the people and context, and evolve and change during a trial, then some of the fundamental assumptions of RCTs may be compromised.

### Phase 2 - testing the theoretical propositions

The seven trials included in phase two provided support for each main proposition coming from phase 1. In addition, cross-comparison of the cases identified a set of sub-domains related to each proposition.

Support for proposition 1 - the importance of context in understanding the mechanism and generalisability of complex interventions - was highly evident and spread evenly across all trials (see Table 5). Clearest and most consistent support was identified in relation to the impact of trial context. However, cross-comparison of the studies generated a reconceptualisation of context as consisting of four, rather than the original three, key contextual domains identified in the original exploratory case. In addition to personal, organisational and trial contexts, it was clear that development of the intervention and operationalisation of it in practice depended on a conceptualisation of the nature, scale, and cause (including stability of the cause) of the underlying problem that the intervention was designed to address. This was therefore referred to as the ‘problem context’.

**Table 5:** Support for proposition 1 - the context within which complex interventions occur is crucial to understanding how that intervention works and how it might be generalisable

**Findings in support of proposition**	**Evidence source**						
	Trial 1	Trial 2	Trial 3	Trial 4	Trial 5	Trial 6	Trial 7
1. Complex interventions (to a greater extent than simple interventions) may require or result in changes in care delivery and therefore demand the involvement and commitment of practitioners and participants within practice settings to make them happen.	✓✓	✓	✓	✓	✓✓	✓✓	✓✓
2. Complex interventions are shaped or co-constructed by aspects of context. Indeed, it seems that the context of a complex intervention may in fact be considered to be a part of that intervention.	✓✓	✓✓	✓		✓✓	✓✓	✓
3. The problem context encompasses:							
· The nature and stability of the scale, distribution and causal mechanism of the problem that the intervention is designed to address. These can change over time and in relation to changes in personal and organisational contexts.	✓✓	✓✓		✓✓	✓✓		✓✓
4. The personal context encompasses:							
· Factors related to the practitioners involved - perceptions of relevance and interest in the intervention, skills, motivation, beliefs, preferences, affinity for intervention, ability to fit it in.	✓✓	✓	✓✓	✓✓	✓✓	✓	✓✓
· Relationships between the practitioner and the participant may become sufficiently important that it becomes a mechanism of action that may facilitate or hinder the effectiveness of the intervention.		✓✓	✓	✓✓	✓✓	✓✓	✓
5. The organisational context encompasses:							
· Organisation of services, managerial support, practicality of delivering interventions, staff availability, venue and timing.	✓✓	✓✓	✓✓	✓			✓✓
6. The trial context encompasses:							
· Personal and interpersonal factors related to the researcher(s) - beliefs and preferences, commitment, role in trial, relationships with practitioners and participants, background and allegiances.	✓✓	✓✓	✓✓	✓✓	✓✓	✓✓	✓✓
· Real and perceived knock-on effects of the intervention on the practice setting.	✓		✓	✓✓	✓✓	✓	✓✓

✓ = some evidence (one instance); ✓✓ = strong evidence (more than one instance); no check/tick = no specific evidence.

There was evidence from a number of trials that the problem context was not only crucial to the success of the intervention but also the utility of the trial results, and thus generalisability. In each study there had been a generally implicit assumption of the cause or nature of the problem, which had then informed the solution - the intervention. However, throughout some of the trials the nature, scale and cause of the problem appeared to shift (with varying and sometimes unknown impact on its size and frequency). For example, in one trial, the intervention was designed for implementation during the phase in which patients were on the waiting list for cardiac bypass surgery. However, during the trial, waiting list targets and fast track initiatives were introduced through policy change, and this meant that the opportunity to deliver the intervention was shortened for many participants. As the researcher explained,

"‘… that was really what crucified the trial because people just didn’t get the interventions they were supposed to get, and I couldn’t recruit certain people, you know, so the whole trial just changed. The outcome of it changed completely, because I couldn’t do the analysis that was originally planned.’"

In examining the evidence further it became apparent that a major cause of the instability of the problem context lay in two further factors: the interdependence of the problem, personal, organisational, and trial contexts, and their own susceptibility to change over time. Consequently, a change in a single factor in one contextual domain had implications for other contextual domains. An example of this ‘ripple effect’ was evident in several trials [29] (see Table 6, finding 4).

**Table 6:** Support for proposition 2 - complex interventions evolve and change

**Findings in support of proposition**	**Evidence source**						
	Trial 1	Trial 2	Trial 3	Trial 4	Trial 5	Trial 6	Trial 7
1. Standardising complex interventions is challenging, because interventions may frequently be inseparable from contexts and contexts themselves are rarely static.	✓✓	✓✓	✓✓	✓✓	✓✓		✓✓
2. Researchers generally have a theory of how the intervention and trial should work but this is not always made explicit. Theories of how an intervention actually should or does work can also change as contextual information becomes more apparent.	✓✓	✓✓	✓	✓✓	✓✓	✓✓	✓✓
3. Relationships between people involved in complex intervention trials evolve and change thereby changing the intervention.	✓	✓			✓	✓	
4. Complex intervention trials have a knock-on effect on the practice context, which may then lead to further evolution and change in the intervention.	✓✓	✓		✓✓	✓		✓✓

✓ = some evidence (one instance); ✓✓ = strong evidence (more than one instance); no check/tick = no specific evidence.

The PI of the carers’ trial described an already existing climate of dissatisfaction amongst carers of people with schizophrenia towards the service provided. Service reorganisation and managerial change took place during the trial, resulting in further dissatisfaction as well as varying attitudes, enthusiasm and engagement from practitioners who were asked to recruit carers.

"‘They [staff] really kept contacting us saying please we want to take part but they were told they couldn’t … they moved geographical area and there was redistribution of staff, some staff left, new staff came in. It caused all sorts of issues and it delayed the period for recruitment and it also delayed the period for the intervention … some of these contextual things are not planned for, they happen during the study … One service was threatened with closure and carers were furious because it meant they would have to travel. They were so angry about services and at the time we were trying to recruit them into the study.’"

In addition to these organisational changes, practitioners were perceived to be ‘choosing’ carers who were likely to benefit from the intervention (see discussion re gatekeeper role), as the PI explained,

"‘They were almost giving them out to who they thought would benefit. In some, what they were making a decision about who would get most benefit and which it would be disruptive to so we had to keep meeting with them saying that wasn’t, you know, it wasn’t about choosing who to distribute to. We tried to explain that everyone had to have an equal chance of making the decision themselves. That was really, really difficult and I’m still not convinced that it came across.’"

A key effect that appeared to mediate this intercontextual dependence was the potential for the individuals and organisation involved to operate as a self-regulating system. For example, in the sleep and exercise trials, as seen in the quotes below, participants in the control arm were perceived to have been motivated to participate in their own interventions. Indeed, trial data showed that patients in the control group engaged in more hours of exercise than the intervention group: -

"‘The control group got better all by itself … was it the Hawthorne effect? Did they start looking at interventions to rectify the problem because they knew they had to wait six months [for a staggered intervention?]"

"‘The control people wanted to be, I thought they would be relieved to be in the control group but weren’t, so they were always very disappointed.’"

Another important aspect of the ‘problem context’ was shown in the sleep trial, where it was clear that participants taking part in the trial were, by nature, different from those who might seek the intervention in a real life setting, and that this inevitably influenced engagement with the intervention:

"‘People who come to the normal clinic have approached a health professional saying “I need help”. I had approached these families, and said “I recognise you have a problem, would you like to take part in this trial and come to this clinic?”. So it was on a very different footing, they hadn’t asked for the help, they were offered it … I think that may have tipped the balance for some of them.’"

Support for proposition 2 - that complex interventions tend to evolve over time - was identified across all trials and stemmed from four sub-domains (see Table 6): first, the frequent inability to meaningfully separate the intervention itself from the changing contexts within which it was embedded; second, the changing understanding of the researchers regarding the perceived cause underlying the problem and thus intervention; third, changes in the relationships between and among practitioners and researchers upon which the intervention may be dependent; and fourth, that the intervention and trial can have a knock-on effect on the practice setting (organisational and problem contexts) which then influence or constrain the intervention being delivered. Strongest cross-trial support was found for the first two sub-domains, while least frequent and evident support existed for the third.

These findings suggested that the distinction between context and intervention may be more conceptually than practically meaningful. Although documentation defined the intervention in terms of a distinct set of events, PIs frequently talked in terms of processes and mechanisms in a more expansive and inclusive manner rather than regarding the intervention as a distinct ‘thing’. Indeed, this highlighted a potentially important distinction between documentation and discussion. Documentation tended to define the intervention in narrower terms and refer to events and sequences. However, interviews with PIs revealed a far more nuanced understanding of the intervention and how it should work. Initially, much of this remained tacit but emerged throughout the interview process after reflection. It appeared, however, that this more sophisticated understanding had not emerged simply as a result of the medium of the interview, but had existed during the trial, often developed as a result of operationalizing the trial protocol. As one researcher said,

"‘If you’re being strict about the way you do the trial then I agree, you shouldn’t really change anything, but I think it’s very hard if you get to the middle of something like a three-year trial and you can see that things aren’t working, it’s … I mean I felt like should I be getting in touch with the funding body and saying look this isn’t working, we ought to be doing something different, and everybody was saying “oh no, you can’t do that, they’ll take all our money away”.’"

Often, interventions themselves differed to those that had originally been planned, or were adapted along the way to improve compliance. Several interventions were designed to include a specific number of ‘sessions’ with a practitioner, but the minimum and maximum number, nature and duration of sessions actually received was extremely variable and this variability had not been pre-specified. Sometimes, interventions had been conceptualised or operationalised differently at the beginning. In one case, an independent assessor of tape recorded cognitive behavioural therapy (CBT) intervention sessions had commented that ‘what was delivered wasn’t precisely what he thought should have been delivered, it was sort of acceptable for one model but not acceptable for a model that he proposes’. In another, the PI wrote in a six-month report; ‘the distinction between the two interventions provided in this study may not be as pronounced as originally intended’.

Support for proposition 3 - that some of the assumptions underpinning RCTs may be compromised for trials of complex interventions - was found to depend on four sub-domains, each of which had varying support across the trials (see Table 7). These sub-domains included: the impact of contextual factors on the RCT; the difficulty of standardisation; the potential inability to meaningfully separate intervention from control given some contextual commonality upon which they may be dependent; and the potential for changes in organisational contexts to differentially impact on the intervention and control groups. Given the earlier indications of the importance of context, it was unsurprising that the strongest and most consistent support related to sub-domain one. However, support for the other domains was evident in a spectrum of trials. PIs acknowledged the principles underpinning RCTs, but demonstrated that in reality, these were highly challenging to maintain. Assumptions were often made about the mechanisms between cause and effect, and strict adherence to the protocol without attention to context meant that important details were often obscured. As one insider on a cluster trial commented,

"‘We were targeting the intervention at one group and gathering the information from another, I think that was laying us open to finding nothing, which was what happened … I think that was fairly evident actually, even at the beginning, but I don’t think enough people noticed it, put it that way. And they were so tied up with the idea … that they didn’t actually stop and think are we actually going to see any change here. And it was patently obvious that we weren’t from the word go.’"

**Table 7:** Support for proposition 3 - the assumptions of the RCT method are less relevant to trials of complex interventions

**Findings in support of proposition**	**Evidence source**						
	Trial 1	Trial 2	Trial 3	Trial 4	Trial 5	Trial 6	Trial 7
1. The contextual factors identified create a number of challenges for RCTs of complex interventions. Tensions exist between the methodological ideals of the RCT method and the reality of evaluating complex interventions within a real life setting and producing useful and meaningful evidence about that intervention.	✓✓	✓✓	✓✓	✓✓		✓✓	✓✓
2. Particular challenges exist in relation to achieving objectivity and standardisation. Personal contexts challenge the concepts of objectivity and equipoise, and all aspects of the reality of clinical practice can interfere with attempts to standardise interventions and protocols.	✓✓	✓	✓	✓✓	✓		
3. The greater the dependence of the intervention and control arms of a trial on the SAME organisational and personal contexts the greater the likelihood that the distinction between the intervention and control will become blurred - contextual commonality.		✓✓		✓	✓✓	✓	✓
4. Changes in the organisational context can differentially impact on the delivery, and thus potentially on the effectiveness, of the intervention and the control.		✓✓			✓✓		

✓ = some evidence (one instance); ✓✓ = strong evidence (more than one instance); no check/tick = no specific evidence.

Although least frequent support was identified in relation to the differential impact of changes in context on the intervention and control groups, in practice this may be extremely important and previously unacknowledged. For example, the degree of practitioner confidence, skill, familiarity and engagement with a trial appears to be central to the delivery of complex interventions and to the differentiation between control and experimental interventions, but this is rarely studied or reported. Indeed, PIs revealed that their RCTs had not always produced useful information that would actually assist in the implementation of similar interventions:

"‘I think this study would have benefited from having a more qualitative element in it as well … at the end of the day, I would want a bit more detail about what factors would make participants engage in the intervention … there’s a lot more to it, I think, than this particular methodology."

"‘I feel there is a need for qualitative data to colour in a lot of the problems, I mean it’s good to evaluate, but it’s also good to use these kinds of experiments as a way of finding out the problems that are going to be involved in implementing an intervention into real life.’"

However, it was clear that even when interventions had not ‘worked’ and RCTs had not delivered the evidence that researchers had hoped for or expected, the trial had often, over time, acted as a catalyst to changes in attitude or organisation of care, which was perceived as a good thing:

"‘It’s changed a lot for the better, I mean it’s still by no means everybody … but certainly a much higher proportion [are now engaging in the intervention]."

"Feedback from the nurses is that a lot of them are using it in practice now. It’s changed the way they see clients.’"

## Discussion

### Summary of findings

We identified a set of characteristics and dimensions that may help describe complex interventions more fully (see Table 8). These dimensions exist and evolve in dynamic contexts that cannot merely be viewed as the inert backdrop for the delivery of the intervention, but are part of the intervention. Pawson *et al*. (2005) describe interventions as ‘leaky’ [22], although they are based on particular theories and given particular titles, they are then ‘delivered in a mutating fashion shaped by refinement, reinvention and adaptation to local circumstances’ (p23). This ‘evolution’ may have unintended consequences such as blurring the distinction between intervention and control, and certain aspects of the intervention ‘slipping’. What matters is less that this occurs (indeed, it can be anticipated for many interventions simply because different people will deliver it) but that it is known, understood and reported.

**Table 8:** Key dimensions of complex interventions


1. Principles/purpose/function - what the intervention is designed to do
2. Target of intervention - is this direct or indirect (that is, does intervention depend on different people/steps to have an impact on target)
3. Structure and architecture - what elements are included and how are they put together (that is, components, steps, order)
4. Theory behind the links between target, structure and purpose (that is, links between 1, 2 and 3)
5. Crucial/central components or defining features (take note, may be an interaction or encompassing element of the bundled intervention)
6. Level of flexibility/tailoring and at what level (that is, tailored to individual, to practitioner, to clinical area)
7. Degree of active participation required from patients/target group
8. Factors that may encourage participation, compliance or uptake from patient/target group
9. Dependence on healthcare professionals - level of input required and at what stages/steps of intervention
10. Number of healthcare professionals required - discrepancies between them
11. Essential characteristics/attributes of healthcare professionals necessary for intervention - skills, knowledge, time, resources, affinity or preference for intervention
12. Location(s) - single, multiple, mixed, home/hospital/primary care, across boundaries
13. Time span - overall timing of intervention, including relationship between recruitment and intervention start/finish, relationship between intervention and timing of illness or critical event, relationship between intervention and outcome measurement
14. Timing of intervention components within structure/architecture of intervention - that is, duration, intervals between, number of sessions
15. Organisational pre-requisites - practitioners, settings, organisational support, organisational structure.

We identified empirical evidence to support the impact of personal, problem, organisation and trial contexts on the running of all trials studied. Importantly, particular elements of these trials appeared to become essential to the effectiveness of the interventions but sometimes changed over time, resulting in varying levels of challenge to the standardisation, control and objectivity of the trial. The nature of the complexity within and around interventions was often embodied in health practitioners’ experience and investigators’ tacit knowledge about the intervention and its implementation. There is a need to explore ways of making these explicit and public. This would help us understand what really contributed to trial outcomes and is essential for replication and use in clinical settings, and for further research [39].

### Discussion of findings in relation to the literature

#### Taxonomies of complexity

Our findings suggest that a taxonomy of complexity may be useful in reflecting different types of contextual influences in trials of complex interventions. Some work in this area has already begun, led primarily by behavioural scientists/health psychologists, categorising behaviour change interventions, techniques and theories [40]. The taxonomy developed by Schulz *et al*. organises the essential features of interventions into two broad categories: 1) treatment delivery characteristics (including mode of delivery, materials, location, schedule, scripting, sensitivity to participant characteristics, interventionist characteristics, adaptability and treatment implementation) and 2) intervention content (treatment content strategies and mechanisms of action) [25].

Intervention taxonomies have a number of benefits, including the highlighting of elements of an intervention that need to be addressed prior to study implementation through pilot work; the recording of intervention details so that analyses can take these into account; and the overall benefits to intervention science [25]. The clear role of what we call contextual factors suggests that trials of some types of complex interventions may be particularly vulnerable to threats to internal validity such as standardisation and intervention fidelity, and that this should be studied explicitly during the trial. Given the varying dependence of the intervention on the personal, problem and organisational contexts within which it is delivered, it is also likely that complex interventions will vary in their generalisability to settings that differ in some degree to that of the original trial [41]. The effectiveness of some interventions may be context resilient (that is, be retained across diverse settings); while others may be highly context sensitive (that is, effectiveness may be lost quickly when applied to different settings or even patient groups). Indeed, recent studies have endorsed one of our own findings, that the characteristics and experiences of the population taking part in a trial may differ in important ways from the ‘real’ population who would normally take up the intervention [41]. Others have found that policy changes can dramatically change the recruitment context in which trials take place, for example, the introduction of a national smoking ban in public places during a trial of a smoking cessation intervention [42]. Our case study adds support to the findings of others suggesting that such taxonomies can be used across multiple trials to identify critical components of interventions and their contexts, and to assess their actual and potential relationship to outcomes [25,43].

#### Widening theoretical eclecticism for the development of complex interventions

Both the 2000 and 2008 versions of the MRC framework highlight the importance of a theoretical basis for the intervention [1,7]. Indeed, given that standardisation of the manifest operational characteristics of the intervention may prove impossible, the best definition of the intervention may in fact be the theory that underpins it.

Since the majority of complex interventions have a behavioural component, theory is often required from the behavioural and social sciences, particularly health psychology. However, given the importance of organisational contexts as evidenced in our study, it is likely that theory from other disciplines may be appropriate, in particular organisational and management theories. Research in the area of social interventions, health promotion and public health that rely on ‘theories of change’ [44] already suggests that when complex behavioural interventions are created, judgments are being made about the ways in which organisations and contexts interact. Indeed, the first task in such theory of change approaches is often to interview the intervention developers to make the assumptions and theory fully explicit. Alternatives to such theory of change approaches are increasingly appearing, including RE-AIM [45], intervention mapping [46] and logic modelling [47].

#### Attitudes and behaviour of practitioners or researchers

Our study showed that practitioners’ roles in research and in their organisation largely shaped the process of the trial and the context within which it operated. First, recruitment and delivery of the intervention depended on their enthusiasm for the intervention and evaluation. A 2006 systematic review of barriers to participation in cancer trials found that clinicians’ concerns about study design and intervention risks and benefits undermined their support for trials [48]. Practitioners sometimes consider complex interventions and trials to be the same thing; therefore, if they do not wish to be involved in providing the intervention, they also reject the trial [49,50]. Clinicians may also reject a complex intervention because of concerns of how it will fit in with their normal practice. All this means that clinicians may employ a gate-keeping role. However, while the literature has explored barriers to recruitment [48], it has not examined in any detail the problem of gate-keeping by clinicians. Many attempts to improve recruitment to RCTs have tended to concentrate on ways of improving the information and support given to potential participants rather than to potential gate-keepers [51,52], and this needs to be addressed.

Once on board, practitioners’ enthusiasm for a trial cannot be assumed to be constant. Research has found that members of staff need to be involved from the beginning, asked for their opinions, and constantly updated on trial progress to maintain their enthusiasm [53]. A systematic review of factors limiting clinician participation in trials found that barriers to clinician participation included time constraints, lack of staff, concern about the impact on doctor-patient relationships, concern for patients, loss of professional autonomy, difficulty with consent procedures and an insufficiently interesting question [54]. A later review confirmed the importance of a clinically relevant research question and good communication [55].

However, practitioners’ enthusiasm for an intervention may make the reality of randomisation particularly challenging [56] and it is important that trialists provide evidence of randomisation integrity. As our findings revealed, practitioners often have ways of compensating control patients by giving them more information, which may encourage contamination between the randomised groups [57]. Indeed, Schulz (1995) argues that ‘RCTs are anathema to the human spirit’ and that once practitioners ‘are engaged in a trial they may find it too difficult to maintain a dispassionate stance’ [58]. We found that practitioners and researchers experienced emotional, practical and philosophical difficulties with adopting the objective perspective required by quantitative research. Other studies have confirmed such conflicts, showing, for example, that blinding can be associated with poorer recruitment [59] and that researchers and practitioners experience a number of challenges to maintaining fidelity in RCTs, particularly related to the balance they need to strike between research and clinical roles [60].

#### Reporting and reflexivity

As mentioned earlier, for complex intervention trials to have an impact on practice they need to provide decision makers - whether at policy or practice level - with sufficient information on the operation and findings of the trial that they can assess validity, reliability, benefit and replicability. Our findings suggest that current reporting standards such as CONSORT [61] are likely to be insufficient to inform clinical and policy judgments. Two issues are important here. First, relevant variables that might aid judgments about internal and external validity need to be reported; and second, sufficient depth and detail with regard to each of those variables is required.

While taxonomies of differing complex interventions may eventually generate new reporting standards, it is likely that the very complexity and idiosyncratic nature of such trials and settings means that relying on reporting standards alone is insufficient. Threats to internal and external validity are likely to emerge that have never and could never have been foreseen and are not listed on reporting standards. Our data suggest that researchers, at least in the past, have tended to be reticent in articulating and reporting the influence of contextual factors on the delivery of interventions within the conduct of their trials, because the RCT has largely been viewed as a controlled and standardisable method, which is acontextual.

Between 2004 and 2010, developments and debates have led to much more open acknowledgement of the challenges of (and some solutions for) conducting RCTs of complex interventions [7,8,62]. A cultural change in reporting is also required; one which regards honest and open reporting as a symbol and requisite of quality in any critical appraisal. Lack of reporting such issues for trials of complex interventions should rightly be regarded as a sign of poor quality, inadequate monitoring and data collection. Managing methodological quality across highly diverse settings has always been a central challenge for qualitative research [63]. We would, therefore, suggest that the concept of ‘reflexivity’ developed in relation to qualitative research, could usefully be applied to all types of research [64-66]. Reflexivity requires the researcher to explicitly acknowledge how researchers’ motivations, personal experiences and inside knowledge of healthcare can affect the way in which they approach the conduct of their research. Finlay and Gough (2003) have set out five variants of reflexivity that could serve as useful guidelines to explore in the conduct and reporting of any research [67]:

‘Five variants of reflexivity’ Finlay and Gough (2003).

1) Reflexivity as introspection - reflection on own experiences and how they relate to the research being conducted. The researcher’s own experiences may influence the research subject, methods chosen, and interpretation of results.

2) Reflexivity as intersubjective reflection - researchers explore the mutual meanings involved within the research relationship. For example, our finding show that researchers were affected by the experiences of research participants, and sometimes this caused them discomfort, particularly when their participants were going through difficult periods of illness.

3) Reflexivity as mutual collaboration - research as a co-constituted account. Our findings illustrate how researchers developed relationships with participants and clinical staff, which affected the nature of data collected and the commitment of parties to the trial.

4) Reflexivity as social critique - recognises the power imbalance between researcher, practitioner, and participant. Our findings illustrated that researchers and practitioners do have preconceived ideas about whether or not the intervention would suit certain types of people. We also noted that gate-keeping by practitioners did exist. Both of which suggest a power imbalance between the actors (researchers, participants and practitioners) involved in research.

5) Reflexivity as ironic deconstruction - recognising ambiguities and multiplicities of meaning. Our researchers’ accounts show that most did not think the results of the trial portrayed the whole picture, and that there were a number of ambiguities and uncertainties about how interventions had worked or not worked, which remained unspoken.

The application of the concept of ‘reflexivity’ to quantitative research would promote transparency around the conduct and reporting of RCTs, which may encourage more realistic accounts of the trial context. This would enable researchers to learn from others’ mistakes, anticipate potential sources of bias, and barriers to recruitment. However, we also note that the tools that encourage reflexivity may, in some circumstances, become part of the intervention rather than a component of the evaluation. For example, one study concluded that conducting a process evaluation alongside a RCT effectively acted as a formative mechanism of feedback to practitioners, encouraging them to reflect on the process of implementing the intervention, and thus subtlety change the intervention [68]. However, this approach may be extremely important to understanding the complexity, flux and contextual variation that inevitably occurs in real life situations [62].

### Strengths and limitations

To our knowledge, this is the first multiple case study of a spectrum of trials of complex interventions. The two-phase design enabled a detailed analysis of insiders’ perspectives of a single trial to inform further exploration of the context of several different trials. This provided an opportunity to use the advantages of the case study approach, through exploration, explanation and theory development.

However, the explanatory phase of the study relied heavily on PIs’ perspectives and would have been strengthened by interviews with other stakeholders, including participants. Additionally, some of the trials sampled were small and were conducted by relatively novice researchers, and most took place in a single site. Although this could potentially explain or limit the generalisability of our findings, 1999 and 2006 analyses of individual trials have revealed very similar issues, illustrating the importance of this work [69,70].

### The way forward?

Trials that take a more pragmatic design approach may provide a solution to the problem of evaluating complex interventions [69,71]. Instead of trying to test the efficacy of an intervention under ideal, experimental conditions, ‘pragmatic trials are designed to find out about how effective a treatment actually is in routine, everyday practice’ [66]. Because of this, results are seen to be more generalisable. However, there may be a trade-off between internal validity (exemplified by the explanatory trial) and external validity (exemplified by the pragmatic trial) and here internal validity could be compromised to ensure generalisability [72], although this remains to be shown empirically. Furthermore, it cannot always be assumed that pragmatic trials really do reflect the real world. Our findings suggest that the very fact that a complex intervention trial is being conducted changes everyday practice making the trial context different from the real world.

Routinely conducting process evaluations alongside pragmatic trials may help. Conceptual models that provide interpretive frameworks for evaluations have been developed such as the Normalization Process Theory [73]. This framework asks what people do to make the complex intervention workable and to integrate it in practice. However, collecting process data can raise additional difficulties [27,68,70]. Researchers, for example, may perceive that an intervention is not working well and then be faced with the dilemma of whether to act on it before the end of the trial [74].

As suggested by the 2008 MRC framework, there are a variety of ways in which trials can be modified to take account of complexity and contextual variation in healthcare practice, including alternative designs such as cluster, stepped wedge or preference designs. Lessons learned from conducting community and public health trials could be usefully applied to trials that take place in more acute settings, where aspects of context are less often considered. Articulating the contextual dimensions of complex interventions using a taxonomy, and incorporating process evaluation (and publishing their protocols), monitoring and reporting of particular threats to validity throughout the trial would improve transparency. Consideration of how the CONSORT extension for complex intervention trials, or the CONSORT statement itself, could be modified to support better reporting of contextual information is also warranted. Finally, it must be recognised that the randomised controlled trial is not appropriate or meaningful for all research questions, and there are many other evaluation approaches that are also important for health improvement [62].

## Conclusion

Our findings add support to the growing interest in the influence of context on trials of complex interventions. Rather than considering context as of background interest, this study illustrates that contextual factors shape or co-construct complex interventions and, therefore, cannot be considered separate from those interventions. Context includes the influence of individuals, teams, organisational structures and cultures, resources, leadership styles and relationships [75]. This suggests that evaluations need to incorporate multiple methods, multiple sources and multiple perspectives if they are to reflect the context of practice adequately.

## Abbreviations

CBT: cognitive behavioural therapy; MRC: Medical Research Council; NLED: nurse-led trial of early discharge following axillary clearance surgery for breast cancer; PI: principal investigator; RCT: randomised controlled trial.

## Competing interests

The authors declare that they have no competing interests.

## Authors’ contributions

MW conceived and designed the study, carried out data collection and analysis and drafted the manuscript. BW and JT participated in all aspects of the study as PhD supervisors. BW and MW conducted further data analysis and literature reviewing to prepare the manuscript. JC and ST contributed to the writing of the paper. All authors read and approved the final manuscript.
